# Effects of auditory rehabilitation with cochlear implant on tinnitus prevalence and distress, health-related quality of life, subjective hearing and psychological comorbidities: Comparative analysis of patients with asymmetric hearing loss (AHL), double-sided (bilateral) deafness (DSD), and single-sided (unilateral) deafness (SSD)

**DOI:** 10.3389/fneur.2022.1089610

**Published:** 2023-01-12

**Authors:** Heidi Olze, Manuel Christoph Ketterer, Dominik Péus, Sophia Marie Häußler, Lynn Hildebrandt, Stefan Gräbel, Agnieszka J. Szczepek

**Affiliations:** ^1^Department of Otorhinolaryngology, Head and Neck Surgery, Charité - Universitätsmedizin Berlin, Corporate Member of Freie Universität Berlin and Humboldt Universität zu Berlin, Berlin, Germany; ^2^Department of Otorhinolaryngology-Head and Neck Surgery, Faculty of Medicine, Medical Center, University of Freiburg, Freiburg, Germany; ^3^Department of Otorhinolaryngology, University of Oldenburg, Oldenburg, Germany; ^4^Department of Otorhinolaryngology, Skull Base Center, University Medical Center Hamburg-Eppendorf, Hamburg, Germany

**Keywords:** auditory rehabilitation, cochlear implant, tinnitus, asymmetric hearing loss, double-sided deafness, single-sided deafness

## Abstract

**Introduction:**

Auditory rehabilitation with a cochlear implant (CI), in many cases, positively impacts tinnitus. However, it is unclear if the tinnitus-related benefit of CI is equal for patients with various indications for CI. Therefore, this study aimed to determine differences in tinnitus prevalence and distress, health-related quality of life, subjective hearing, perceived stress, and psychological comorbidities between patients diagnosed with asymmetric hearing loss (AHL), single-sided (unilateral) deafness (SSD), and double-sided (bilateral) deafness (DSD) before and six months after cochlear implantation.

**Methods:**

One hundred-one CI candidates were included in this prospective study (39 AHL patients, 23 DSD patients, and 39 SSD patients). The patients completed questionnaires measuring tinnitus distress, health-related quality of life, subjective hearing, perceived stress, and psychological comorbidities before and 6 months after CI.

**Results:**

The prevalence of tinnitus in the entire cohort (80.2% before CI) decreased 6 months after CI to 71.3%. The DSD group had the lowest tinnitus prevalence at both time points. The degree of tinnitus-induced distress decreased significantly in all three groups after CI. Differences in quality of life, subjective hearing, and psychological comorbidities between the groups at the study onset disappeared after CI. Significant correlations existed between anxiety, depression, and tinnitus distress in AHL and SSD but not in DSD patients before and after CI.

**Discussion:**

Our results demonstrate significant differences between the three groups of CI candidates, which might affect the implantation outcome. These differences suggest a need for personalized psychological counseling during the auditory rehabilitation process, focusing on anxiety and depressive symptoms for SSD and AHL patients.

## 1. Introduction

Auditory rehabilitation with cochlear implants (CI) is an optional treatment for profoundly hard of hearing or deaf children and adults (1). Cochlear implantation improves general hearing abilities, speech perception, and sound localization in patients with asymmetric hearing loss (AHL) (2), single-sided (unilateral) deafness (SSD) (3), and double-sided (bilateral) deafness (DSD) (4). Accumulating evidence suggests that the beneficial effects of implantation extend beyond the main indication (improvement of auditory abilities) and can positively impact cognition (5–7), health-related quality of life (6, 8), and comorbid symptoms such as depression or anxiety (9). Additionally, the predominantly positive influence of implantation on tinnitus presence and tinnitus-induced distress is well-documented (9, 10), although the exact mechanism of this phenomenon is still not fully understood.

Tinnitus is a perception of sound in the absence of an external acoustic stimulus (11). It is a symptom that can be induced by various pathological mechanisms, including cochlear deafferentiation seen in CI candidates with profound unilateral or bilateral sensorineural hearing loss (11, 12). The loss of signal from the periphery contributes to neuroplastic changes in the central auditory system, resulting in the activation of the primary auditory cortex. Tinnitus percept can but does not have to be bothersome.

Previous research demonstrated a significant impact of tinnitus on auditory rehabilitation outcomes in patients implanted with CI. In implantees with SSD and DSD types of deafness, the degree of tinnitus-induced distress correlated negatively with the hearing-related quality of life (13). Similar results were obtained in a different sample of implanted SSD and DSD patients, indicating that tinnitus can predict the overall hearing-related quality of life after implantation (14). In addition, several other studies have shown that auditory rehabilitation with CI generally reduces tinnitus burden (15–17). The relationship between tinnitus and regaining auditory abilities after implantation is heterogeneous. On the one hand, tinnitus can impact the outcomes of CI by creating a challenge in CI programming and negatively influencing patients' satisfaction with CI (18, 19). On the other hand, auditory rehabilitation with CI frequently reduces tinnitus (20–22), but in some cases, it might induce tinnitus or worsen its burden (23, 24). In recent years, we have shown a variety of benefits of cochlear implantation, including tinnitus reduction, in three distinct groups of patients with AHL (25), SSD (26), and DSD (27). These studies supported the notion of auditory rehabilitation with CI restoring binaural hearing leading to improvement of hearing abilities and tinnitus burden in all three groups.

Although there is a wealth of research on tinnitus-related outcomes of cochlear implantation, only a few studies compared the tinnitus-related and other outcomes between implantees based on various indications for CI. Hence, here, we performed comparative analyses and evaluated the impact of rehabilitation with CI on tinnitus and tinnitus-induced distress between three groups of hard-of-hearing patients: AHL, SSD, and DSD. Additionally, we explored possible differences between the three groups regarding health-related quality of life, auditory abilities, perceived stress, and presence and grade of comorbid anxiety and depressive symptoms.

To address the above issues in detail, we posed three research questions. (1) “*Does the tinnitus prevalence and severity differ between AHL, SSD, and DSD patients) before and 6 months after cochlear implantation?”* (2) “*Do the health-related quality of life, auditory abilities, perceived stress, and presence and grade of comorbid anxiety and depressive symptoms differ between AHL, SSD, and DSD patients before and 6 months after CI?”* (3) “*Does the strength of the relationship between the variables (tinnitus-induced distress, health-related quality of life, auditory abilities, perceived stress, and presence and grade of comorbid anxiety, and depressive symptoms) vary between AHL, SSD, and DSD patients before and 6 months after CI?”*.

## 2. Material and methods

The present study was approved by the Ethics Committee of Charité Universitaetsmedizin-Berlin (**EA2/030/13**). The investigations were conducted according to the principles expressed in the Declaration of Helsinki, and all subjects gave written informed consent. Data were prospectively collected from a cohort of 101 patients (54 women and 47 men) included in the study between 2013 and 2021. The inclusion criteria comprised age (18 years of age or older), confirmed diagnosis of AHL (39 patients), DSD (23 patients), and SSD (39 patients), and qualification to the cochlear implantation program (see Table 1 for detailed characteristics of the study population). All the DSD patients were implanted sequentially, and the median time between the implantations was 14.9 months (Table 1). These patients completed the questionnaires before the first and 6 months after the second implantation.

**Table 1:** Descriptive statistics of the cohort.

	**Whole cohort**	**AHL**	**DSD**	**SSD**
*n*	101	39	23	39
Women (*n*)	54	20	8	26
Men (*n*)	47	19	15	13
Age in years (mean) ± SD	58.7 ± 14.1	61.7 ± 13.2	57.3 ± 12.3	54.0 ± 16.0
Age range in years (min.-max.)	21.5–80.6	26.2–79.7	41.9–79.7	21.5–80.6
Deafness duration in years (median and range)	18 (1–67)	22 (1–67)	23 (1–63)	3 (1–55)
For DSD patients only: time in months between 1st and 2nd CI (median and range)	n.a.	n.a.	14.9 (6.0–55.0)	n.a.

AHL, asymmetric hearing loss; DSD, double-sided (bilateral) deafness; SSD, single-sided (unilateral) deafness; SD, standard deviation; CI, cochlear implant; n.a., not applicable.

The diagnosis of AHL was based on the presence of severe to profound sensorineural hearing loss in the poorer ear [average hearing loss ≥70 dB SPL (sound pressure level)] and audiometric hearing loss of ≤60 dB SPL up to 4 kHz and >30 dB SPL in at least one frequency up to 4 kHz in the better ear (25). The diagnosis of DSD was made based on bilateral sensorineural severe or profound hearing loss with speech recognition ≤40% in the Freiburg Monosyllabic Test in quiet and with a hearing aid using 65-dB SPL (28). The diagnosis of SSD was made based on the presence of severe to profound sensorineural hearing loss in the poorer ear (average hearing loss ≥70 dB SPL) and normal hearing in the better ear. The hearing level in the better ear could not exceed the hearing loss threshold of 30 dB in 500, 1,000, 2,000, and 4,000 Hz, as per pure tone audiometry (26).

**Figure 1:**
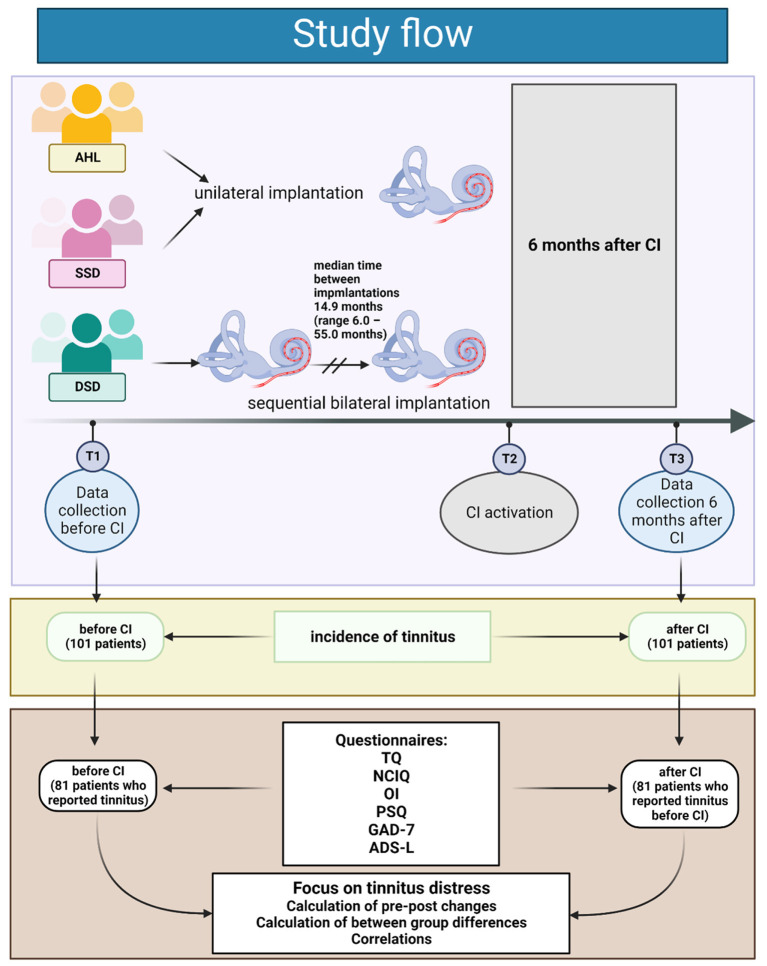
The data was collected from all patients at the study baseline (T1). Only the patients with tinnitus were included in further analysis. T2 indicates the time at which the patients in all groups had their CI activated. The second data set was collected at T3 (six months after activation of the cochlear implant). Created with BioRender.com.

All 101 patients were included in the analysis of tinnitus incidence. Thirty-three AHL, 16 DSD, and 32 SSD patients who reported tinnitus at the study onset were included in further analyses of pre-post changes in tinnitus burden and other variables tested and correlations between tinnitus distress and other variables. The study flow is presented in Figure 1.

### 2.1. Questionnaires

#### 2.1.1. Tinnitus questionnaire (TQ)

Several psychometric instruments were developed to test the degree of tinnitus-induced burden. One of these instruments is Tinnitus Questionnaire (TQ), developed by Hallam et al. (29), and since 1994, used in its validated German version (30). The TQ measures the degree of tinnitus-induced distress and consists of 52 items assigned to 6 scales (tinnitus-induced emotional distress, cognitive distress, intrusiveness, auditory perceptual difficulties, sleep disturbances, and somatic complaints) (30). Scores can range from 0 to 84, and the results are interpreted as a degree of tinnitus-induced distress: a 1st-degree burden represents a score of 0–30 points; 2nd-degree to 31–46 points; 3rd-degree, 47–59 points; and 4th-degree, 60 to 84 points.

A therapeutically relevant system for tinnitus classification based on the total TQ score was developed (31). That system uses the TQ cutoff score of 47 to split the patients into a compensated group (meaning the patients are habituated to the tinnitus sound) and decompensated group (indicating that tinnitus is not habituated and induces suffering in the affected patients) (32). According to German guidelines, there is no therapeutic need for patients with compensated chronic tinnitus, while psychological or psychosomatic treatment is recommended for patients with decompensated chronic tinnitus (33). The goal of that therapy is a reduction of tinnitus distress.

#### 2.1.2. Oldenburg inventory (OI)

The subjective hearing was measured with the short version of OI (34) that has 12 questions and three scales: (1) listening in a quiet setting (questions 1, 3, 5, 7), (2) listening with background noise (4, 6, 8, 11, 12), (3) directional listening (2, 9, 10). Responses to each of the 12 questions were rated 1 to 5 points. The higher the total score, the better the subjective hearing.

#### 2.1.3. Nijmegen cochlear implantation questionnaire (NCIQ)

The NCIQ estimates patients' quality of life before and after cochlear implantation (35). NCIQ contains six scales and consists of 60 items. The scales reflect the following domains: physical domain (NCIQ1 basic sound perception, NCIQ2 advanced sound perception, NCIQ3 speech production); psychological domain (NCIQ4 self-esteem); social domain (NCIQ5 activity limitations and NCIQ6 social interactions). The NCIQ scores are normalized to percentages. The higher the total score, the better the health-related quality of life.

#### 2.1.4. General anxiety disorder 7 questionnaire (GAD-7)

GAD-7 measures the frequency and degree of anxiety within the 2 weeks before taking the test (36). Seven items are measured and scored based on patients' responses, using the following scale: 0 = not at all; 1 = on some days; 2 = more often than every other day; 3 = almost every day. A sum of the scores provides a value for estimating the degree of the burden presented by fear (small, mild, medium, or severe). The answer scores range between 0 and 21. The higher the score, the greater the anxiety.

#### 2.1.5. General depression scale (ADS)

The ADS uses 20 items to measure depressive symptoms (presence, degree, and length of depressive episodes) within a week before the test (37). The total score ranges from 0 to 60; a score of 23 is considered the cutoff value for major depression.

#### 2.1.6. Perceived stress questionnaire (PSQ)

The German version of PSQ measures a subjective perception of stress (38). The short version of the PSQ used in the present study consists of 20 items (4 subscales: worries, tension, joy, and demands). Each scale can have values from 0 to 1. A score above 0.45 represents a moderate stress level, and above 0.6 represents a high-stress level.

### 2.2. Statistical analysis

The data were analyzed using the German version of IBM SPSS Statistics 27 (IBM Deutschland GmbH, Ehningen, Germany). Descriptive statistics determined the variables' means, standard deviation, and minimum and maximum values. Most data did not have a normal distribution as per the Kolmogorov-Smirnov test; thus, non-parametric tests were used to calculate the pre-post differences, the between-group differences, and correlations. Contingency tables were used to determine the proportion of tinnitus in subgroups. Wilcoxon test for paired samples was applied for the within-groups calculation of changes before-after implantation. For the between-group comparison, we used Kruskal–Wallis test with Bonferroni correction. The relationships between the variables were tested using Spearman correlation. *P*-values <0.05 were considered statistically significant.

## 3. Results

### 3.1. Prevalence of tinnitus and clinically relevant compensation-decompensation status before and after CI

Before CI, the prevalence of tinnitus in the entire cohort was 80.2%; in the AHL group, 84.6%; in the DSD group, 69.6% and in the SSD group, 82.1% (Table 2). After CI, 71.3% of the entire cohort reported having tinnitus (71.8% in the AHL group, 52.2% in the DSD group, and 82.1% in the SSD group).

**Table 2:** Contingency table showing tinnitus prevalence and degree of tinnitus-induced distress before and after CI for each group and the whole sample.

	**AHL (39 patients)**	**DSD (23 patients)**	**SSD (39 patients)**	**Total (101 patients)**
Tinnitus-positive patients before CI	33 (84.6%)	16 (69.6%)	32 (82.1%)	81 (80.2%)
Tinnitus-positive patients after CI	28 (71.8%)	12 (52.2%)	32 (82.1%)	72 (71.3%)
Tinnitus-negative patients before and after CI	4 (10%)	6 (26%)	4 (10%)	14 (13.9%)
Patients who developed tinnitus after CI	2 (5%)	1 (4%)	3 (8%)	6 (5.9%)
Pateints who reported tinnitus vanishing after CI	5 (13%)	5 (22%)	3 (8%)	13 (12.9%)
Patients with decompensated (not habituated) tinnitus before CI (TQ>47)	9 (23.1%)	3 (13.0%)	9 (23.1%)	21 (20.8%)
Patients with decompensated (not habituated) tinnitus after CI (TQ>47)	5 (12.8%)	0 (0.0%)	9 (23.1%)	14 (13.9%)

AHL, asymmetric hearing loss; DSD, double-sided (bilateral) deafness; SSD, single-sided (unilateral) deafness; CI, cochlear implantat; TQ, Tinnitus Questionnaire.

Fourteen of 20 patients in the entire cohort who were tinnitus-free before CI (13.9%) remained tinnitus-free after CI, whereas 6 (5.9%) reported post-CI tinnitus. Thirteen patients who reported tinnitus before CI (12.9%) were tinnitus-free 6 months after CI (Table 2). Interestingly, the latter group comprised 22% DSD, 13% AHL, and only 8% SSD patients.

A clinically significant change in tinnitus when using German TQ was determined to be 12 points (39). Improvement of tinnitus by 12 or more points was noted in 41 (50.6%) of all patients [17 AHL patients (51.5%), 12 DSD patients (75.0%), and 12 (37.3%) SSD patients]. No clinical change in tinnitus was seen in 38 entire cohort patients (50.6%). In individual groups, no difference was reported in 16 (48.5%) of the AHL group, 3 (18.8%) of the DSD, and 19 (59.4%) of the SSD group. Tinnitus worsened significantly in one DSD patient and one SSD patient.

Regarding the clinically significant tinnitus compensation (habituation) status, before implantation, 21 patients (20.8%) of the entire cohort had decompensated (not habituated) tinnitus. 6 months after CI, this number decreased to 14 (13.9%) patients. None of the patients in the DSD group reported decompensated tinnitus after CI, and the incidence was roughly equal in the other two groups.

### 3.2. Changes in tinnitus-related distress within the groups 6 months after cochlear implantation

Changes in tinnitus-induced distress measured by TQ and assessed with Wilcoxon Test indicated that 6 months after regaining bilateral hearing, the total TQ scores significantly decreased in all groups (Supplementary Table 1). In addition, the scores of TQ subscales (emotional distress, cognitive distress, intrusiveness, auditory perceptual difficulties) decreased significantly in all groups. The subscales “sleep disturbances” and “somatic complaints” decreased significantly only in the DSD group.

### 3.3. Post-implantation changes in hearing-related variables and psychological comorbidities within the groups

The overall health-related quality of life indicated by a total score of NCIQ (Supplementary Table 1) significantly improved in all three groups after 6 months of auditory rehabilitation (measured with Wilcoxon Test, significance is shown in Supplementary Table 1). However, the improvement in the subscales varied. A significant improvement in NCIQ1 (basic sound perception) was seen in the DSD and SSD but not in the AHL group. A considerable improvement in NCIQ2 (advanced sound perception) was seen only in the DSD group. The DSD and SSD groups significantly improved their NCIQ3 (speech production). The domain NCIQ4 (self-esteem) increased significantly in all three groups after implantation. The NCIQ5 score (social activity limitations) improved in DSD and SSD, whereas the NCIQ6 (social interactions) improved significantly in all three groups.

The subjective quality of sound perception (in quiet, noise, directional and overall) measured by the Oldenburg Inventory improved significantly (Supplementary Table 1) in all three groups.

Only a few changes were found in perceived stress (PSQ) after CI. The subscale “tension” significantly decreased in the SSD but not in other groups, whereas the subscale “demands” improved significantly but only in the AHL group (Supplementary Table 1).

The GAD-7 score (before and after CI) decreased, indicating a significant decrease in anxiety symptoms in the SSD (Supplementary Table 1) but not in the other groups.

### 3.4. Differences in tinnitus-related distress between the groups before and 6 months after implantation

**Figure 2:**
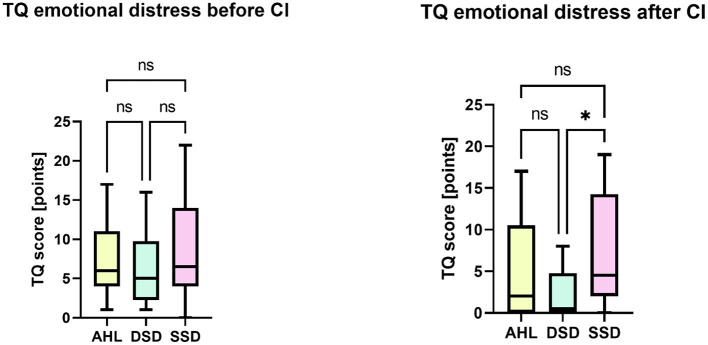
Between-group differences regarding the TQ subscale “emotional impact of tinnitus”. Kruskal-Wallis with Bonferroni correction (^*^p < 0.05) demonstrated that six months after cochlear implantation, the DSD group patients reported significantly less tinnitus-induced emotional distress than the SSD group. ns, not significant.

Kruskal–Wallis test performed for the patients who reported having tinnitus has not indicated significant differences between the AHL, DSD, and SSD groups regarding subscales or the total score of the TQ before CI. However, 6 months after implantation, we found a significant difference between the groups regarding the subscale “emotional distress” [*H* (2) = 7.398, *p* = 0.025]. DSD patients reported significantly less tinnitus-induced emotional distress (*Mdn* 2) than the SSD patients (*Mdn* 5), with Bonferroni-adjusted alpha level *p* = 0.020 (Figure 2).

### 3.5. Between-group differences regarding non-tinnitus variables in patients who reported tinnitus

#### 3.5.1. Health-related quality of life (NCIQ)

We analyzed the differences between the three groups regarding the health-related quality of life (NCIQ), hearing quality (Oldenburg Inventory), perceived stress (PSQ), as well as the grade of depressive (ADS) and anxiety symptoms (ADL) before and after cochlear implantation.

**Figure 3:**
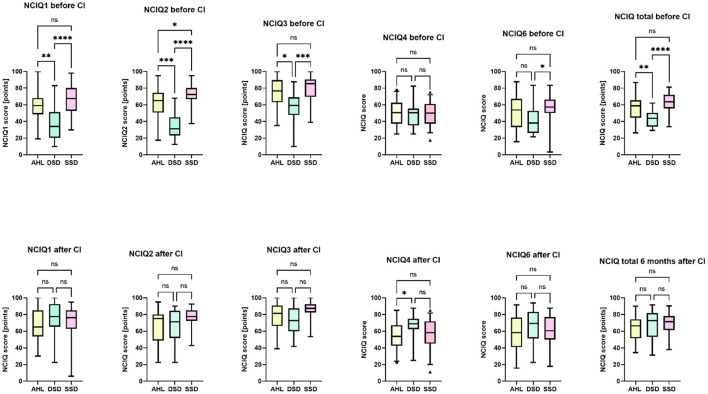
Between-group differences in the health-related quality of life (NCIQ) before and after CI. The Kruskal-Wallis test with Bonferroni- correction indicated significant differences. ^*^p < 0.05. ^**^p < 0.01. ^***^p < 0.001. ^****^p = 0.0. ns, not significant.

Before CI, there were significant differences between the groups regarding the basic sound perception NCIQ1 [*H* (2) = 19.328, *p* = 0.000], advanced sound perception NCIQ2 [*H* (2) = 32.246, *p* = 0.000], speech production NCIQ3 [*H* (2) = 16.929, *p* = 0.000], social interactions NCIQ6 [*H* (2) = 8.095, *p* = 0.017], and the total NCIQ score [*H* (2) = 20.983, *p* = 0.000] (Figure 3). Detailed analysis revealed that the SSD group's health-related quality of life was lower than the other two groups. Kruskal–Wallis test indicated no differences between the groups regarding self-esteem NCIQ4 or social activity limitations NCIQ5.

6 months after CI, there was a difference in the NCIQ4 [*H* (2) = 6.368, *p* = 0.042], reflecting significantly greater self-esteem of DSD patients than in the AHL (but not SSD) group. All groups scored similarly in the rest of the scales and the total NCIQ score.

#### 3.5.2. Oldenburg inventory

**Figure 4:**
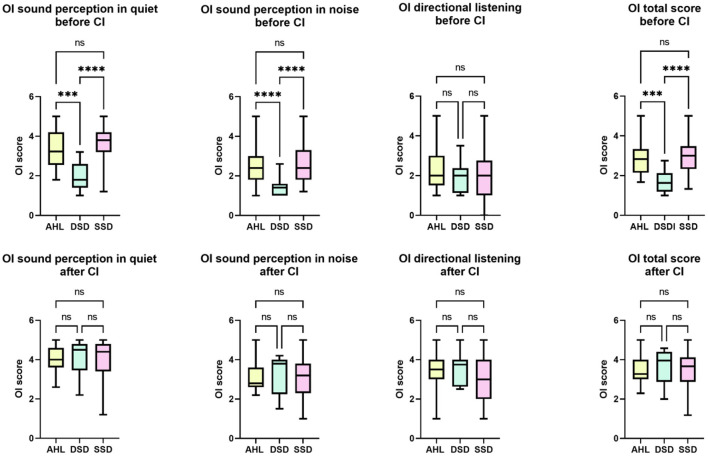
Between-group differences in the self-reported quality of hearing before and after CI. The sound perception in quiet and noise, as well as the total score of the Oldenburg Inventory, differed between AHL and DSD and SSD and DSD but not AHL and SSD before implantation. The DSD group scored poorer than the other two. Six months after the activation of CI, there were no longer differences between the groups. ^***^p < 0.001. ^****^p = 0.0. ns, not significant.

Regarding the OI, there were significant differences between the groups except for directional hearing. The “hearing in quiet” differed significantly between the groups [*H* (2) = 27.154, *p* = 0.000], as did the “hearing with background noise” [*H* (2) = 22.728, *p* = 0.000], and the total OI score [*H* (2) = 24.297, *p* = 0.000], reflecting the presence of profoundly bilaterally hard of hearing patients DSD, who significantly differed from the SSD and AHL. At the same time, no differences were found between SSD and AHL (Figure 4).

6 months after the CI activation (or activation of the second CI for DSD patients), the differences between the groups were no longer found.

#### 3.5.3. Perceived stress (PSQ)

**Figure 5:**
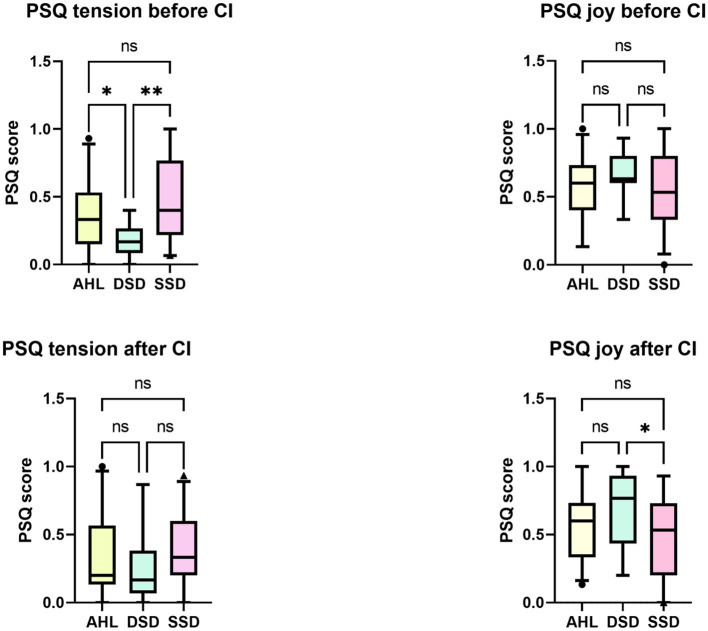
Between-groups differences in the perceived stress (PSQ). At the study onset, the DSD group reported significantly less tension than the AHL and SSD patients. Six months after implantation, there were no longer differences detected with the Kruskal-Wallis test. ^*^p < 0.05. ^**^p < 0.01 after Bonferroni correction. ns, not significant.

Before the activation of CI, there were between-group differences in the subscale “tension” of the PSQ questionnaire (*H* (2) = 10.492, *p* = 0.005). The DSD groups perceived significantly less stress-related tension than the AHL (Bonferroni-adjusted alpha level *p* = 0.047) and the SSD (Bonferroni-adjusted alpha level *p* = 0.004). 6 months after the CI activation, significant differences between the groups were found in the subscale “joy” (*H* (2) = 6.290, *p* = 0.043). The DSD group had higher scores than the SSD group (Bonferroni-adjusted alpha level *p* = 0.037); (see Figure 5).

#### 3.5.4. Anxiety and depression (GAD-7 and ADS-L)

**Figure 6:**
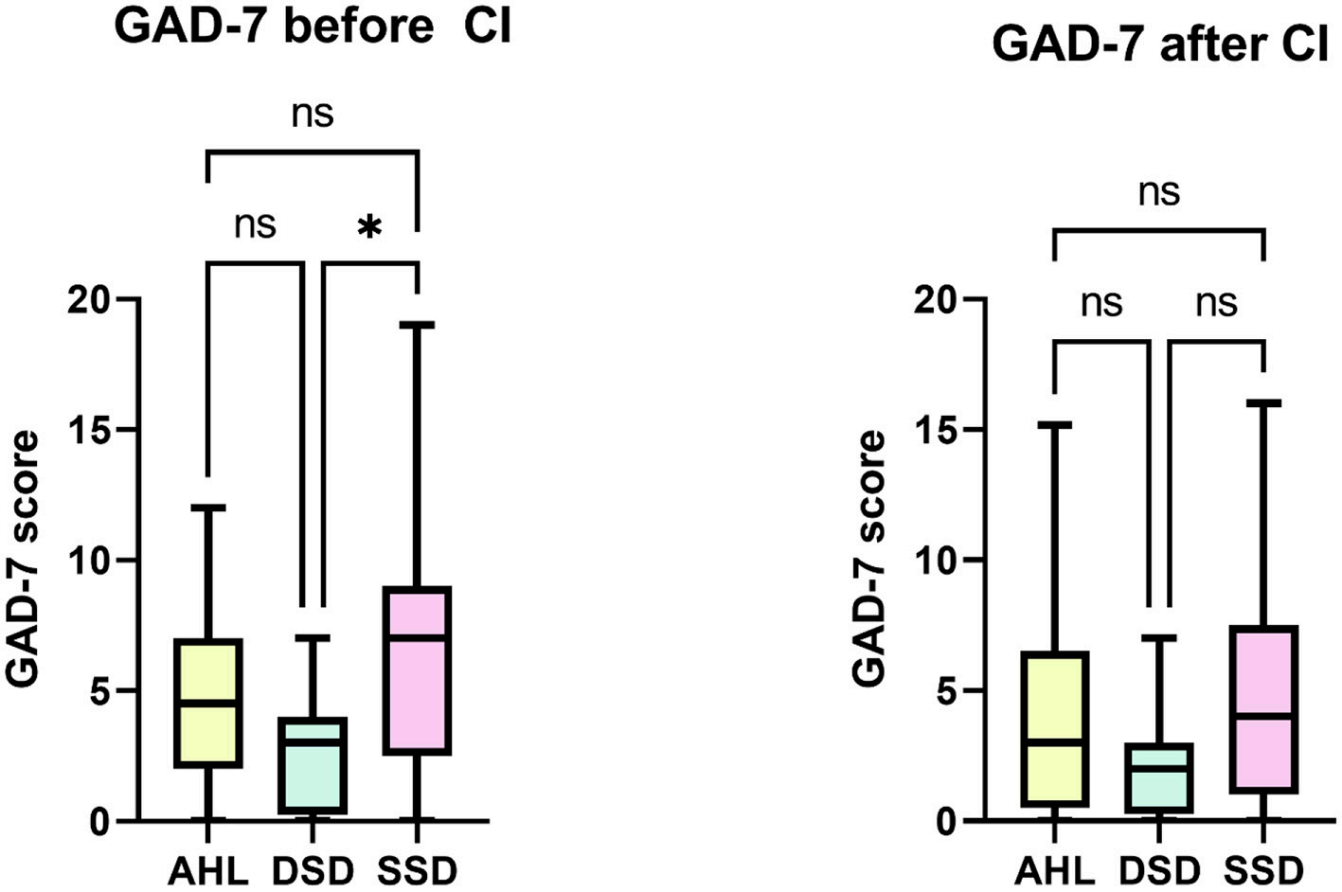
Between-group differences in the anxiety symptoms (GAD-7). Kruskal-Wallis test indicated a significant difference between the DSD and SSD groups at the study onset. After implantation, there were no longer differences between the groups. ^*^p < 0.05; ns, not significant.

The anxiety differed between the groups before implantation (*H* (2) = 9.383, *p* = 0.009). The SSD group scored significantly higher in GAD-7 than the DSD group (Bonferroni-adjusted alpha level *p* = 0.007) but not the AHL group (Figure 6). 6 months after CI, no differences in GAD-7 were found between the groups.

The ADS-L scores did not differ between the groups before or after implantation.

#### 3.5.5. The correlation pattern differs between the groups

Spearman's rank correlation assessed the relationship between tinnitus-induced distress (total score of TQ) and the total scores of health-related quality of life (NCIQ), Oldenburger Inventory (OI), perceived stress (PSQ), anxiety (GAD-7), and depressive symptoms (ADS-L) before and after CI in each group (Table 3).

**Table 3:** The Spearman correlation was computed for the main variables before and after implantation.

			**TQ before**	**NCIQ before**	**OI before**	**PSQ before**	**GAD-7 before**	**ADS-L before**			**TQ after**	**NCIQ after**	**OI after**	**PSQ after**	**GAD-7 after**	**ADS-L after**
AHL	TQ before	r_s_	–						TQ after	r_s_	–					
		*p*	.							*p*	.					
		N	33							n	33					
	NCIQ before	r_s_	−0.532^**^	–					NCIQ after	r_s_	−0.483^**^	–				
		*p*	0.002	.						*p*	0.004	.				
		N	32	32						n	33	33				
	OI before	r_s_	−0.410^*^	0.742^**^	–				OI after	r_s_	−0.331	0.354	–			
		*p*	0.018	0	.					*p*	0.074	0.055	.			
		N	33	32	33					n	30	30	30			
	PSQ before	r_s_	0.233	−0.433^*^	−0.262	–			PSQ after	r_s_	0.436^*^	−0.594^**^	−0.317	–		
		*p*	0.2	0.015	0.148	.				*p*	0.018	0.001	0.107	.		
		N	32	31	32	32				n	29	29	27	29		
	GAD-7 before	r_s_	0.549^**^	−0.568^**^	−0.441^*^	0.631^**^	–		GAD-7 after	r_s_	0.487^**^	−0.423^*^	−0.541^**^	0.601^**^	–	
		*p*	0.001	0.001	0.01	0	.			*p*	0.005	0.016	0.002	0.001	.	
		N	33	32	33	32	33			n	32	32	30	29	32	
	ADS-L before	r_s_	0.306	−0.635^**^	−0.293	0.484^**^	0.530^**^	–	ADS-L after	r_s_	0.467^**^	−0.518^**^	−0.505^**^	0.597^**^	0.744^**^	–
		*p*	0.083	0	0.098	0.005	0.001	.		*p*	0.007	0.002	0.004	0.001	0	.
		N	33	32	33	32	33	33		N	32	32	30	29	32	32
DSD	TQ before	r_s_	–						TQ after	r_s_	–					
		*p*	.							*p*	.					
		N	16							n	16					
	NCIQ before	r_s_	−0.318	–					NCIQ after	r_s_	−0.173	–				
		*p*	0.231	.						*p*	0.521	.				
		N	16	16						n	16	16				
	OI before	r_s_	−0.467	0.646^**^	–				OI after	r_s_	−0.274	0.917^**^	–			
		*p*	0.069	0.007	.					*p*	0.304	0	.			
		N	16	16	16					n	16	16	16			
	PSQ before	r_s_	−0.034	−0.318	0.23	–			PSQ after	r_s_	0.118	−0.361	−0.412	–		
		*p*	0.901	0.23	0.392	.				*p*	0.663	0.17	0.112	.		
		n	16	16	16	16				n	16	16	16	16		
	GAD-7 before	r_s_	−0.650^**^	0.123	0.202	0.071	–		GAD-7 after	r_s_	−0.368	0.095	−0.014	0.649^**^	–	
		*p*	0.006	0.65	0.454	0.793	.			*p*	0.16	0.726	0.96	0.007	.	
		n	16	16	16	16	16			n	16	16	16	16	16	
	ADS-L before		0.007	−0.302	0.07	0.834^**^	0.012	–	ADS-L after		0.066	−0.308	−0.173	0.685^**^	0.434	–
		*p*	0.978	0.256	0.798	0	0.965	.		*p*	0.808	0.245	0.522	0.003	0.093	.
		n	16	16	16	16	16	16		n	16	16	16	16	16	16
SSD	TQ before	r_s_	–						TQ after	r_s_	–					
		*p*	.							*p*	.					
		n	32							n	32					
	NCIQ before	r_s_	−0.636^**^	–					NCIQ after	r_s_	−0.628^**^	–				
		*p*	0	.						*p*	0	.				
		n	32	32						n	32	32				
	OI before	r_s_	−0.360^*^	0.803^**^	–				OI after	r_s_	−0.333	0.569^**^	–			
		*p*	0.043	0	.					*p*	0.063	0.001	.			
		n	32	32	32					n	32	32	32			
	PSQ before	r_s_	0.585^**^	−0.296	−0.122	–			PSQ after	r_s_	0.513^**^	−0.502^**^	−0.346	–		
		*p*	0	0.1	0.508	.				*p*	0.003	0.003	0.052	.		
		n	32	32	32	32				n	32	32	32	32		
	GAD-7 before	r_s_	0.602^**^	−0.298	−0.179	0.762^**^	–		GAD-7 after	r_s_	0.559^**^	−0.599^**^	−0.17	0.727^**^	–	
		*p*	0	0.098	0.326	0	.			*p*	0.001	0	0.351	0	.	
		N	32	32	32	32	32			n	32	32	32	32	32	
	ADS-L before	r_s_	0.648^**^	−0.308	−0.204	0.785^**^	0.733^**^	–	ADS-L after	r_s_	0.625^**^	−0.561^**^	−0.410^*^	0.868^**^	0.758^**^	–
		*p*	0	0.086	0.263	0	0	.		*p*	0	0.001	0.02	0	0	.
		N	32	32	32	32	32	32		N	32	32	32	32	32	32

^**^The correlation is significant (two-tailed) at the 0.01 level.

^*^The correlation is significant (two-tailed) at the 0.05 level.

AHL, asymmetric hearing loss; DSD, double-sided (bilateral) deafness; SSD, single sided (unilateral) deafness; TQ, Tinnitus Questionnaire; NCIQ, Nijmegen Cochlear Implant Questionnaire measuring health-related quality of life; OI, Oldenburg Inventory measuring subjective hearing quality; PSQ, Perceived Stress Questionnaire; ADS-L, General Depression Scale; GAD-7, General Anxiety Disorder 7 Questionnaire; r_s_, correlation coefficient; *p*, probability level; N, number of subjects included in given analysis.

At the study onset, the total TQ score in the AHL and SSD groups correlated positively with anxiety (GAD-7) and negatively with the health-related quality of life scores (NCIQ). In addition, in the SSD patients, we found a positive correlation between the TQ score and depressive symptoms (ADS-L), TQ and perceived stress (PSQ), and a negative correlation between TQ and the OI. In the DSD group, the TQ scores negatively correlated with anxiety levels (GAD-7).

After cochlear implantation, the correlation pattern between TQ and other variables has changed. In the AHL group, relationships between TQ and NCIQ or GAD-7 remained (but the correlation coefficient value decreased). However, the correlation between TQ and OI was no longer detected. Two new positive relationships between TQ and PSQ and TQ and ADS-L were seen, indicating a possible influence of tinnitus on perceived stress and depressive symptoms. No correlations between TQ and any other variable were found in the DSD group. In the SSD group, all correlations remained the same, with one exception: TQ no longer correlated with the Oldenburg Inventory score.

We also analyzed other relationships, the first between NCIQ and other variables. At the study onset, there was a positive correlation between NCIQ and Oldenburg Inventory scores in all three groups. After CI, this correlation was no longer significant in the AHL patients, unlike in DSD and SSD groups. Furthermore, before implantation, we found negative correlations between NCIQ and PSQ, NCIQ and GAD-7, and NCIQ and ADS-L in the AHL group (but not DSD or SSD). These correlations suggest the negative influence of perceived stress, anxiety, and depressive symptoms on health-related quality of life in patients with asymmetric hearing loss. 6 months after CI, these correlations persisted in the AHL group and appeared in the SSD group. Additionally, a negative correlation between NCIQ and ADS-L was present in the SSD group before implantation. This correlation was also present after implantation in the AHL and SSD groups, indicating a negative association between depressive symptoms and the health-related quality of life in AHL and SSD but not DSD patients.

In all three groups, before and after implantation, there was a significant positive correlation between ADS-L and PSQ. The positive correlation between ADS-L and GAD-7 before and after implantation was seen only in the AHL and SSD groups. Finally, PSQ correlated positively with GAD-7 before implantation in the AHL and SSD and after implantation in all three groups of patients.

## 4. Discussion

At the beginning of this study, we posed three research questions. Based on the performed analyses, the answer to our first question is that the tinnitus prevalence does differ between AHL, SSD, and DSD patients. We observed that before implantation, the AHL group had the highest prevalence of tinnitus (84.6%), followed by SSD (82.1%) and DSD (69.6%). Tinnitus prevalence reported in the literature varies between the studies and is sometimes very low [22% (40)], some other times in the middle range such as 65% (41) or 70% (42), and finally as high as 80% (43), 85% (44), or even 90% (45). Unfortunately, none of the above studies provided information about the deafness laterality of the subjects included in that research. More recent studies have delivered information on the prevalence of tinnitus among groups of patients with defined types of deafness. In a sample of 51 DSD patients, 94.1% of CI candidates had preoperative tinnitus (46). In other studies, the prevalence of tinnitus in DSD patients was 42% (21) and 55.8% (47), which is comparable to our results. The majority of data regarding tinnitus incidence in severe and profoundly deaf people originates from the field of cochlear implant, and the subjects included in the research were verified CI candidates.

After cochlear implantation, tinnitus prevalence decreased and differed between the groups. The SSD group remained at the same level as before CI. In that group, three persons reported tinnitus loss after CI, but the other three, originally tinnitus-free, reported tinnitus occurring after implantation. In the other two groups, tinnitus prevalence decreased. A newly induced tinnitus was observed in all groups but with different frequencies (Table 2, 5.9% of the entire cohort). An earlier report determined the prevalence of post-CI tinnitus to be 12.7% in a cohort of 187 implanted DSD patients (48), which is three times more than in our present study. However, in contrast to our study, only the unilaterally implanted DSD patients were included in that sample. Another study found tinnitus induction in 13% of DSD patients 1 year after CI (23). These patients were, however, simultaneously implanted, whereas ours were implanted sequentially. A systematic review published in 2015 determined the prevalence of newly induced tinnitus in implanted DSD patients to be between 0 and 10% (21) and pointed out methodological differences between the studies included in the review, suggesting a need for further studies using uniform design. As for SSD patients, the evidence provided by another systematic review (10) proposed an absence of tinnitus induction in implanted SSD patients. However, only three studies included in that particular sub-analysis of tinnitus prevalence used 6 months of follow-up after CI (49–51). Only one of the 39 patients included in the analysis was tinnitus-free before CI. In contrast, in our study, 18% of SSD patients were tinnitus-free before CI. We failed to identify a survey on tinnitus incidence in AHL patients after CI. Our results and those of others indicate the direction of further research in which standardized pre-CI diagnostics and follow-up conducted in multicentric studies could contribute to providing answers to still-open questions.

The severity of tinnitus is a critical factor negatively affecting auditory function and rehabilitation. In all three groups, tinnitus severity significantly decreased after CI. Nevertheless, the groups differed concerning tinnitus-induced sleep disturbances and somatic complaints, with only DSD patients, but not AHL or SSD patients, having significantly reduced scores after CI. It should, however, be noted that in all the groups, the median values of somatic and sleep complaints were low (Supplementary Table 1). In addition, it should be remembered that DSD patients were implanted twice (sequentially). In that group, there is a known and previously described benefit of the first implantation on tinnitus (21), which could have influenced the final results after the second implantation. Furthermore, a between-groups comparison indicated that after CI, the DSD patients are under significantly less tinnitus-induced emotional distress than the SSD (but not AHL) patients. A recent systematic review found a substantial benefit of cochlear implantation concerning tinnitus for SSD patients (10) and determined that 90% of SSD patients reported decreased tinnitus distress after CI. This finding agrees with the results presented here, despite the short time of data collection (6 months after activation of CI), as we found that 69.7% of SSD patients reported improvement and 9.1% of SSD patients total disappearance of tinnitus (total 79.8%). A recent systematic review supports our findings in the SSD group (10). Another systematic review conducted for the DSD patients also found a benefit of CI regarding tinnitus incidence and a decrease in tinnitus-induced distress (21) but pointed to inadequate evidence of the studies included. Finally, a systematic review analyzing the benefit of CI in AHL and SSD patients identified research on changes in tinnitus-related parameters (e.g., tinnitus loudness or tinnitus-induced distress) but pointed out a significant heterogeneity of studies included and a need for more studies (52).

The answer to our second question is “partially yes.” There were differences in the health-related quality of life, auditory abilities, perceived stress, and presence and grade of comorbid anxiety and depressive symptoms between AHL, SSD, and DSD groups. We first evaluated the changes in subjective hearing and hearing-related activities assessed with NCIQ and OI. At the study onset, we found several between-group differences consistent with the DSD patients having the worse health-related quality of life. That observation agrees with Blue Mountains Hearing Study results, showing a significantly worse quality of life in bilaterally deafened adults than in the unliterally affected persons (53). The only difference between the AHL and SSD groups was in the advanced sound perception (NCIQ2), which was better in the SSD than in AHL patients, confirming our earlier observations (25). 6 months after CI, all domains of NCIQ equalized between the groups. A single significant difference between the groups indicated that DSD patients' self-esteem (NCIQ4) was better than AHL patients. We have previously performed a comparative analysis of similar parameters between the AHL and DSD groups and found that 6 months after implantation, the DSD group had significantly lower NCIQ2 and NCIQ3 scores than the AHL group (27). In the present detailed analyses, unlike before, we included only patients with tinnitus at the study onset. Therefore, our findings support the notion of tinnitus impacting auditory rehabilitation with CI. Since the DSD patients were the most successful group in our study regarding reducing tinnitus incidence and decreasing scores of all tinnitus subscales, one could assume that this reduction had positively influenced the quality of life. However, correlation analyses (discussed below) have not confirmed this hypothesis for the DSD group; therefore, one should seek other explanations. At the study onset, the subjective audiological assessment with Oldenburg Inventory indicated that the DSD patients have worse sound perception in quiet and noise and the total OI score than the AHL or SSD patients. This result is not surprising for the DSD patients, who were bilaterally deaf before the implantation. However, these differences were no longer present 6 months after CI indicating that according to the patient's subjective view, their hearing performance was alike in all groups.

Analysis of psychological comorbidities indicated that before implantation, the subscale “tension” in the perceived stress questionnaire was lower in the DSD group than in AHL or SSD. After implantation, apart from the scores of the subscale “joy” that was higher in the DSD group than in the SSD patients, there were no other between-group differences. Pre-post analysis within groups indicated only minor changes, namely a significant tension decrease in the SSD group and a decrease in demands in the AHL group. Previous research stated improvement of all PSQ subscales after a more extended period of 24 months after CI (54), but the study sample included patients with and without tinnitus. There were no differences in depressive symptoms (ADS-L) levels between the groups before or after CI. There was, however, a difference in anxiety score (GAD-7), being higher in SSD than in the DSD group before CI. The anxiety levels equalized after CI due to a significant decrease in the GAD-7 score within the SSD group. The presence and impact of anxiety on the lives of unilaterally deafened patients were determined in qualitative research (55), confirming our qualitative findings.

The answer to our last question is “yes.” We detected a pattern in relationships between variables that were distinct for a given group. At the study onset, we found positive associations between tinnitus-induced distress (TQ) and anxiety (GAD-7) but only in AHL and SSD groups (Table 3). In the DSD group, we found a negative relationship between the TQ and GAD-7, indicating that increased anxiety correlated with decreased tinnitus-induced distress. Score values and sample size, which are low in the DSD group, can explain this surprising finding (see Supplementary Table 1). In contrast, the scores of the AHL and SSD groups are significantly higher than the DSD (Figure 6). We found a positive correlation between TQ and the SSD group's perceived stress level (PSQ), confirming earlier reports (56). Furthermore, TQ correlated negatively with NCIQ and OI in both AHL and SSD groups. 6 months after CI, in the AHL and SSD groups, we observed the same correlation pattern between TQ and other variables: a positive correlation with PSQ, GAD-7, and ADS-L and a negative correlation with NCIQ. As for the DSD group, no significant correlations were detected between TQ and any other variables, confirming our earlier published observation (57). These results suggest that the number of analyzed CI candidates with DSD and tinnitus should be increased in the future. The analysis should also be performed between the first and second implantation. The results obtained for the SSD and AHL groups imply the need for psychological intervention during auditory rehabilitation. Psychological counseling could help reduce the negative impact of comorbidities on hearing abilities (seen in the correlation) and improve the outcomes of auditory rehabilitation. Lowering the significant negative correlations between comorbid psychological symptoms and NCIQ or OI would likely benefit rehabilitation outcomes.

The lack of correlations between TQ and other variables in the DSD group suggests that these patients do not require additional psychological support to aid their auditory rehabilitation with a second CI. Unlike the AHL and SSD groups, the DSD patients were implanted twice between T1 and T2 (Figure 1). Our present research focused on the analysis after the second implantation in this group; however, it is known that already after the first implantation, DSD patients benefit in terms of quality of life (NCIQ) and subjective quality of hearing (OI) and that this positive change is sustainable (58). Therefore, some of the parameters measured at T3 could have been improved in the DSD group before.

### 4.1. Study limitations

Our study is not free of pitfalls; the first is the small sample size of the subgroups, which could be expanded in a multicenter study or an extended study duration in the future. The second drawback of our work is that the study looked at a relatively short period after cochlear implantation (6 months). To track changes that might have occurred later, we need to prolong the observation time in the future. Finally, the data regarding tinnitus is limited, as it does not contain detailed information about laterality, matching, loudness, tinnitus awareness, and other tinnitus-related variables.

## 5. Conclusions

This study identified differences between three groups of CI candidates (AHL, DSD, SSD) concerning tinnitus, quality of life, subjective hearing, and psychological comorbidities. Most of these differences prevailed before implantation but faded 6 months after CI. Tinnitus prevalence varied among AHL, DSD, and SSD groups before and after CI, being the lowest in the DSD group at both times. Tinnitus distress improved significantly after 6 months of auditory rehabilitation with CI, as did the quality of life and subjective hearing in patients who initially reported tinnitus in all three groups. The differences between AHL, DSD, and SSD groups before CI in the quality of life, subjective hearing, perceived stress, and anxiety symptoms disappeared after CI. We suggest considering several features associated with hearing loss type and the presence or absence of tinnitus when planning auditory rehabilitation with CI. In individual cases, particularly AHL and SSD patients, psychological interventions targeting tinnitus distress and mental comorbidities could indirectly improve the health-related quality of life and subjective hearing of implanted tinnitus patients.

## Data availability statement

The raw data that support the findings of this study are available from the corresponding author HO, upon reasonable request.

## Ethics statement

The studies involving human participants were reviewed and approved by Ethikkommission der Charité–Universitätsmedizin Berlin (EA2/030/13). The patients/participants provided their written informed consent to participate in this study.

## Author contributions

HO: study concept, project supervision, drafting, and revising the manuscript. MK, DP, and SH: data collection, interpretation, and revising the manuscript. LH and SG: data collection and interpretation. AS: data interpretation, drafting and revising the manuscript, and data visualization. All authors contributed to the article and approved the submitted version.
