# Supplementary material for: Effects of auditory rehabilitation with cochlear implant on tinnitus prevalence and distress, health-related quality of life, subjective hearing and psychological comorbidities: Comparative analysis of patients with asymmetric hearing loss (AHL), double-sided (bilateral) deafness (DSD), and single-sided (unilateral) deafness (SSD)
Source: Front Neurol. 2023 Jan 12;13:1089610. doi: 10.3389/fneur.2022.1089610 (PMC9877424; doi:10.3389/fneur.2022.1089610)
Supplement: Supplementary file 1 [file Table_1.DOCX]

**Supplementary Table 1**

Median scores with sample size, range, and SD of individual questionnaires and subscales per group at T1 and T3 (before and six months after cochlear implantation). Only the patients who reported tinnitus before CI were included in the analysis. The two-sided statistical significance of changes (before-after CI) was calculated using the Wilcoxon test.

|  |  | before CI | | | | 6 months after CI | | | | *p* |
| --- | --- | --- | --- | --- | --- | --- | --- | --- | --- | --- |
| group | questionnaire | *n* | Min. | Max. | median | *n* | Min. | Max. | median |  |
| AHL | TQ emotional distress | 33 | 1.00 | 17.00 | **6.0** | 33 | 0.00 | 17.00 | **2.0** | **0.006*** |
|  | TQ cognitive distress | 33 | 0.00 | 13.00 | **6.0** | 33 | 0.00 | 16.00 | **4.0** | **0.009*** |
|  | TQ intrusiveness | 33 | 1.00 | 15.00 | **9.0** | 33 | 0.00 | 14.00 | **4.0** | **0.000*** |
|  | TQ auditory perceptual difficulties | 33 | 0.00 | 14.00 | **5.0** | 33 | 0.00 | 12.00 | **2.0** | **0.000*** |
|  | TQ sleep disturbance | 33 | 0.00 | 8.00 | **2.0** | 33 | 0.00 | 7.00 | **0.0** | 0.076 |
|  | TQ somatic complaints | 33 | 0.00 | 4.00 | **0.0** | 33 | 0.00 | 5.00 | **0.0** | 0.110 |
|  | TQ total | 33 | 1.00 | 63.00 | **28.0** | 33 | 0.00 | 60.00 | **12.0** | **0.000*** |
|  | NCIQ 1 | 32 | 19.00 | 100.00 | **59.0** | 33 | 30.00 | 100.00 | **65.0** | 0.072 |
|  | NCIQ 2 | 32 | 17.50 | 95.00 | **65.0** | 33 | 22.50 | 95.00 | **75.0** | 0.150 |
|  | NCIQ 3 | 32 | 35.00 | 100.00 | **76.8** | 33 | 38.89 | 100.00 | **81.3** | 0.433 |
|  | NCIQ 4 | 32 | 25.00 | 77.50 | **50.3** | 33 | 22.50 | 85.00 | **53.8** | **0.020*** |
|  | NCIQ 5 | 32 | 0.00 | 80.60 | **49.9** | 33 | 27.50 | 87.50 | **56.9** | 0.074 |
|  | NCIQ 6 | 32 | 15.60 | 87.50 | **53.8** | 33 | 15.60 | 91.70 | **58.3** | **0.014*** |
|  | NCIQ Total | 32 | 26.30 | 86.70 | **59.0** | 33 | 34.40 | 90.10 | **66.5** | **0.019*** |
|  | OI quiet setting | 33 | 1.80 | 5.00 | **3.2** | 30 | 2.60 | 5.00 | **4.0** | **0.000*** |
|  | OI noise interference | 32 | 1.00 | 4.40 | **2.2** | 30 | 2.20 | 4.80 | **2.8** | **0.000*** |
|  | OI directional listening | 33 | 1.00 | 4.00 | **2.0** | 30 | 1.00 | 4.50 | **3.5** | **0.000*** |
|  | OI total | 32 | 1.67 | 4.58 | **2.5** | 30 | 2.30 | 4.60 | **3.3** | **0.000*** |
|  | PSQ worries | 32 | 0.00 | 0.80 | **0.7** | 29 | 0.00 | 1.00 | **0.1** | 0.329 |
|  | PSQ tension | 32 | 0.00 | 0.93 | **0.3** | 29 | 0.00 | 1.00 | **0.2** | 0.628 |
|  | PSQ joy | 32 | 0.13 | 1.00 | **0.6** | 29 | 0.13 | 1.00 | **0.6** | 0.943 |
|  | PSQ demands | 32 | 0.00 | 0.87 | **0.3** | 29 | 0.00 | 1.00 | **0.2** | **0.017*** |
|  | PSQ total | 32 | 0.03 | 0.78 | **0.4** | 29 | 0.03 | 0.95 | **0.3** | 0.710 |
|  | Anxiety (GAD7) | 33 | 0.00 | 12.00 | **5.0** | 32 | 0.00 | 15.17 | **3.0** | 0.531 |
|  | Depression (ADS-L) | 33 | 1.00 | 29.00 | **13.0** | 32 | 0.00 | 35.00 | **11.0** | 0.479 |
| DSD | TQ emotional distress | 16 | 1.00 | 16.00 | **5.0** | 16 | 0.00 | 8.00 | **0.5** | **0.006*** |
|  | TQ cognitive distress | 16 | 0.00 | 12.00 | **4.5** | 16 | 0.00 | 13.00 | **1.0** | **0.003*** |
|  | TQ intrusiveness | 16 | 0.00 | 16.00 | **8.0** | 16 | 0.00 | 12.00 | **1.0** | **0.007*** |
|  | TQ auditory perceptual difficulties | 16 | 0.00 | 13.00 | **5.0** | 16 | 0.00 | 10.00 | **1.5** | **0.001*** |
|  | TQ sleep disturbance | 16 | 0.00 | 7.00 | **1.5** | 16 | 0.00 | 3.00 | **0.0** | **0.019*** |
|  | TQ somatic complaints | 16 | 0.00 | 4.00 | **1.0** | 16 | 0.00 | 1.00 | **0.0** | **0.010*** |
|  | TQ total | 16 | 2.00 | 62.00 | **23.5** | 16 | 0.00 | 44.00 | **5.0** | **0.003*** |
|  | NCIQ 1 | 16 | 10.00 | 83.00 | **34.0** | 16 | 22.50 | 100.00 | **77.5** | **0.001*** |
|  | NCIQ 2 | 16 | 12.50 | 67.86 | **31.3** | 16 | 22.50 | 90.00 | **71.3** | **0.002*** |
|  | NCIQ 3 | 16 | 10.00 | 87.50 | **59.4** | 16 | 41.67 | 100.00 | **72.9** | **0.003*** |
|  | NCIQ 4 | 16 | 25.00 | 82.50 | **50.4** | 16 | 25.00 | 87.50 | **68.8** | **0.005*** |
|  | NCIQ 5 | 16 | 20.00 | 70.00 | **40.0** | 16 | 27.50 | 95.00 | **64.4** | **0.005*** |
|  | NCIQ 6 | 16 | 21.43 | 83.30 | **38.2** | 16 | 22.50 | 93.75 | **69.4** | **0.003*** |
|  | NCIQ Total | 16 | 29.20 | 62.04 | **43.8** | 16 | 31.47 | 91.53 | **72.8** | **0.001*** |
|  | OI quiet setting | 16 | 1.00 | 3.20 | **1.8** | 16 | 2.20 | 5.00 | **4.5** | **0.000*** |
|  | OI noise interference | 16 | 1.00 | 2.60 | **1.4** | 16 | 1.50 | 4.20 | **3.8** | **0.001*** |
|  | OI directional listening | 16 | 1.00 | 3.50 | **2.0** | 16 | 2.50 | 5.00 | **3.8** | **0.000*** |
|  | OI total | 16 | 1.00 | 2.75 | **1.6** | 16 | 2.00 | 4.58 | **4.0** | **0.000*** |
|  | PSQ worries | 16 | 0.00 | 0.40 | **0.2** | 16 | 0.00 | 0.73 | **0.1** | 0.258 |
|  | PSQ tension | 16 | 0.00 | 0.40 | **0.2** | 16 | 0.00 | 0.87 | **0.2** | 0.609 |
|  | PSQ joy | 16 | 0.33 | 0.93 | **0.6** | 16 | 0.20 | 1.00 | **0.8** | 0.289 |
|  | PSQ demands | 16 | 0.00 | 0.67 | **0.2** | 16 | 0.00 | 0.93 | **0.1** | 0.650 |
|  | PSQ total | 16 | 0.07 | 0.40 | **0.2** | 16 | 0.00 | 0.70 | **0.2** | 0.605 |
|  | Anxiety (GAD7) | 16 | 0.00 | 7.00 | **3.0** | 16 | 0.00 | 7.00 | **2.0** | 0.431 |
|  | Depression (ADS-L) | 16 | 2.00 | 22.00 | **9.00** | 16 | 3.00 | 29.00 | **8.0** | 0.551 |
| SSD | TQ emotional distress | 32 | 0.00 | 22.00 | **6.5** | 32 | 0.00 | 19.00 | **4.5** | **0.000*** |
|  | TQ cognitive distress | 32 | 0.00 | 16.00 | **5.0** | 32 | 0.00 | 16.00 | **2.5** | **0.004*** |
|  | TQ intrusiveness | 32 | 0.00 | 16.00 | **8.0** | 32 | 0.00 | 15.00 | **6.0** | **0.000*** |
|  | TQ auditory perceptual difficulties | 32 | 0.00 | 14.00 | **5.0** | 32 | 0.00 | 12.00 | **2.5** | **0.000*** |
|  | TQ sleep disturbance | 32 | 0.00 | 8.00 | **1.0** | 32 | 0.00 | 7.00 | **0.0** | 0.858 |
|  | TQ somatic complaints | 32 | 0.00 | 6.00 | **1.0** | 32 | 0.00 | 6.00 | **0.0** | 0.580 |
|  | TQ total | 32 | 2.00 | 77.00 | **24.5** | 32 | 0.00 | 70.00 | **14.0** | **0.000*** |
|  | NCIQ 1 | 32 | 30.00 | 98.00 | **67.5** | 32 | 6.00 | 95.00 | **76.3** | **0.011*** |
|  | NCIQ 2 | 32 | 37.50 | 95.00 | **72.5** | 32 | 42.86 | 92.50 | **77.5** | 0.141 |
|  | NCIQ 3 | 32 | 38.90 | 100.00 | **85.6** | 32 | 53.57 | 100.00 | **87.5** | 0.155 |
|  | NCIQ 4 | 32 | 17.50 | 75.00 | **51.3** | 32 | 11.11 | 85.00 | **58.3** | **0.032*** |
|  | NCIQ 5 | 32 | 25.00 | 75.00 | **52.5** | 32 | 21.87 | 100.00 | **60.6** | **0.006*** |
|  | NCIQ 6 | 32 | 3.57 | 83.33 | **57.3** | 32 | 17.86 | 87.50 | **60.6** | **0.016*** |
|  | NCIQ Total | 32 | 33.60 | 81.30 | **63.7** | 32 | 38.00 | 90.42 | **69.39** | **0.001*** |
|  | OI quiet setting | 32 | 1.20 | 5.00 | **3.8** | 32 | 1.20 | 5.00 | **4.4** | **0.027*** |
|  | OI noise interference | 32 | 1.20 | 4.50 | **2.4** | 32 | 1.00 | 4.80 | **3.2** | **0.003*** |
|  | OI directional listening | 32 | 0.00 | 3.50 | **2.0** | 32 | 1.00 | 5.00 | **3.0** | **0.000*** |
|  | OI total | 32 | 1.33 | 4.18 | **2.9** | 32 | 1.18 | 5.00 | **3.6** | **0.007*** |
|  | PSQ worries | 32 | 0.00 | 0.93 | **0.2** | 32 | 0.20 | 1.33 | **0.3** | 0.801 |
|  | PSQ tension | 32 | 0.07 | 1.00 | **0.4** | 32 | 0.00 | 0.93 | **0.3** | 0.060 |
|  | PSQ joy | 32 | 0.00 | 1.00 | **0.5** | 32 | 0.27 | 0.93 | **0.5** | 0.207 |
|  | PSQ demands | 32 | 0.00 | 1.00 | **0.3** | 32 | 0.00 | 0.93 | **0.2** | 0.092 |
|  | PSQ total | 32 | 0.03 | 0.95 | **0.3** | 32 | 0.06 | 0.93 | **0.3** | 0.313 |
|  | Anxiety (GAD7) | 32 | 0.00 | 19.00 | **7.0** | 32 | 0.00 | 16.00 | **4.5** | **0.036*** |
|  | Depression (ADS-L) | 32 | 0.00 | 46.00 | **13.0** | 32 | 0.00 | 46.00 | **14.0** | 0.992 |
